# Manipulating nonlinear exciton polaritons in an atomically-thin semiconductor with artificial potential landscapes

**DOI:** 10.1038/s41377-023-01268-2

**Published:** 2023-09-08

**Authors:** Yuan Luo, Quanbing Guo, Xinyi Deng, Sanjib Ghosh, Qing Zhang, Hongxing Xu, Qihua Xiong

**Affiliations:** 1 State Key Laboratory of Low-Dimensional Quantum Physics, Department of Physics, Tsinghua University, Beijing, 100084 China; 2 Wuhan Institute of Quantum Technology, Wuhan, 430206 China; 3 School of Materials Science and Engineering, Peking University, Beijing, 100871 China; 4 Beijing Academy of Quantum Information Sciences, Beijing, 100193 China; 5 School of Physics and Technology, Center for Nanoscience and Nanotechnology, and Key Laboratory of Artificial Micro- and Nano-structures of Ministry of Education, Wuhan University, Wuhan, 430072 China; 6 Frontier Science Center for Quantum Information, Beijing, 100084 China; 7 Collaborative Innovation Center of Quantum Matter, Beijing, China

**Keywords:** Polaritons, Nonlinear optics

## Abstract

Exciton polaritons in atomically thin transition-metal dichalcogenide microcavities provide a versatile platform for advancing optoelectronic devices and studying the interacting Bosonic physics at ambient conditions. Rationally engineering the favorable properties of polaritons is critically required for the rapidly growing research. Here, we demonstrate the manipulation of nonlinear polaritons with the lithographically defined potential landscapes in monolayer WS_2_ microcavities. The discretization of photoluminescence dispersions and spatially confined patterns indicate the deterministic on-site localization of polaritons by the artificial mesa cavities. Varying the trapping sizes, the polariton-reservoir interaction strength is enhanced by about six times through managing the polariton–exciton spatial overlap. Meanwhile, the coherence of trapped polaritons is significantly improved due to the spectral narrowing and tailored in a picosecond range. Therefore, our work not only offers a convenient approach to manipulating the nonlinearity and coherence of polaritons but also opens up possibilities for exploring many-body phenomena and developing novel polaritonic devices based on 2D materials.

## Abstract (web summary)

We demonstrate the manipulation of nonlinear polaritons and their prolonged coherence by creating fully deterministic potential wells with the lithographic mesas to trap polaritons in a monolayer WS_2_ microcavity.

## Graphical abstract

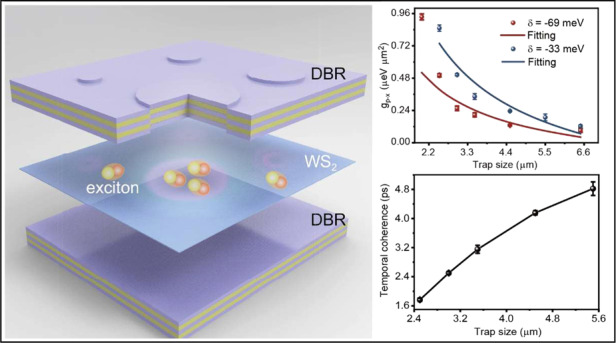

## Introduction

Exciton polaritons, hybrid quasiparticles caused by the strong exciton-photon coupling, constitute a unique prototype for studying non-equilibrium physics and quantum photonic applications, such as superfluidity, Berezinskii–Kosterlitz–Thouless transition, and quantum simulations traditionally in cryogenic conditions^1–4^. Atomically thin transition-metal dichalcogenides (TMDs), as exceptional semiconductors with room-temperature operations, have received much attention due to their fascinating valleytronics features and strong exciton resonance^5–7^. In recent years, exciton polaritons in monolayer TMDs have been widely investigated by integrating with various cavities^8–12^ and revealed many interesting phenomena^13–17^. In view of the hybrid nature, polaritons possess a nonlinear optical response governed by the excitonic constituent^18^, which makes them an excellent candidate for accessing nonclassical light and quantum networks^4,19–22^. Nevertheless, in TMD microcavities, the overall nonlinear interaction strength of polaritons can be insignificant compared to that of other wide-bandgap semiconductors^23–25^. Considerable effort has been devoted to improving the nonlinear interactions, for instance, by resorting to 2*s* states^26^, trion^27^, and moiré^28^ or dipolar^29^ excitons. However, these excitons quickly dissipate at elevated temperatures and then destroy the strong coupling condition. Thus, achieving an appropriate combination of strong nonlinearity together with the thermal stability of the TMD polaritons is highly sought after for realistic polariton-based integrated devices^17,30^.

Confining polaritons with a well-controlled potential is an effective means for the manipulation of the polariton system, harnessing their interaction strength and new quantum phase of quasiparticles^31–33^. Recently, locally trapping the polaritons in TMD cavities was achieved via random disorders probably due to strain or air gaps involved in sample preparation. Motional narrowing^34^, macroscopic coherence^35^, and enhanced relaxation^36^ were observed due to the spatial confinement of polaritons. These encouraging findings are important steps in the development of TMD polaritons and also imply a promising possibility that TMDs can be preferential room-temperature substitutes for traditional semiconductors. Nevertheless, the uncontrollable potential sites in previous literature hinder the further exploration of both fundamental physics and applications^3,30,37^. Considering the extremely small Bohr radius and a sub-micrometer transport length of excitons^38^, confining polaritons through the photonic component seems to be more practical, for example, by concave cavities^8,39^. However, the complex operations in open cavities are difficult to make compatible with chip-scale polaritonic circuits and the nonlinear response of polaritons in these traps remains unrevealed, which is of great importance for functional polaritonic devices^40,41^.

Here, we create fully deterministic potential wells *via* the lithographic mesas to trap polaritons^25,42,43^ in a monolayer WS_2_ microcavity. The confinement strength is tailored by designing the sizes of cylindrical mesa structures from 6.5 μm to 2 μm, as evidenced by the increased energy differences between the discretized polariton quantum states. We have systematically studied the polariton nonlinearity by nonresonant power-dependent measurements, and find that the polariton–exciton interaction dominates the observed spectral shift and can be increased by about six times through improving spatial confinement at room temperature. The first-order correlation measurements prove the prolonged coherence for the trapped polaritons and the wide-range tunability of the temporal coherence based on the nonlinear-interaction-related dephasing. Thus, our work strongly indicates the feasibility of controlling polariton properties in TMD microcavities by engineering artificial potential wells and establishes the foundation for future integrated polaritonic devices operating at room temperature^33,44,45^.

## Results

**Fig. 1:**
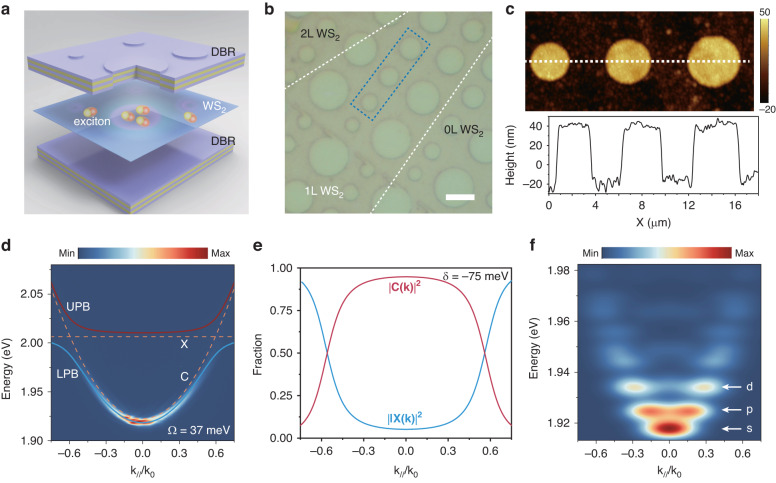
Sample structure and polariton dispersion. **a** Schematic of monolayer WS_2_ embedded in two DBRs with cylindrical mesa structures. The exciton polaritons are trapped in the artificial confinement potential. **b** Optical microscope image of the sample. The dashed white lines outline the monolayer WS_2_ boundary. The scale bar corresponds to 5 μm. **c** AFM image (upper panel) of exemplary mesas selected from the blue dashed region in (**b**). The cross-section profile of the mesas is plotted in the lower panel. **d** Angle-resolved photoluminescence dispersion collected from a planar microcavity. The dashed lines describe the bare exciton (*X*) and microcavity modes (*C*). The red (blue) line represents the upper (lower) polariton branch. **e** Fraction coefficient ${|X(k)|}^{2}$ for the excitonic part (blue lines) and ${|C(k)|}^{2}$ for the photonic part (red lines) in the lower polariton branch extracted from the coupled oscillator model. **f** Theoretically calculated polariton dispersion for the mesa structure with a diameter of 4.5 μm. The energies of *s*, *p*, an*d d* states are indicated by the white arrows

In our experiment, the structure of TMD microcavity is schematically shown in Fig. 1a. The monolayer WS_2_ locates around the anti-node in the *λ*/2 cavity formed by two distributed Bragg reflectors (DBR) to ensure a strong exciton-photon coupling. The mesa structures are fabricated by patterning the polymethyl methacrylate (PMMA) spacer using electron-beam lithography before the deposition of top DBR (see Materials and Methods section). The optical image of the whole microcavity is displayed in Fig. 1b. To achieve variable spatial confinement, the diameters of the cylindrical mesa structures are designed between 2 μm and 6.5 μm. The distance of neighboring pillars is kept at more than 2.5 μm to avoid near-field interaction between them and guarantee far-field resolvability. To estimate the potential depth provided by the mesa, the surface morphologic images of several typical mesas (blue region in Fig. 1b) are measured by atomic force microscopy (AFM), as shown in the upper panel of Fig. 1c. We find that the average depth is about 59 nm, which corresponds to a potential depth of 183 meV, much larger than the exciton linewidth. To characterize the optical quality of the mesa cavity, the angle-resolved reflectivity was measured (Fig. S1) and the quality factor is calculated to be about 900.

Considering the spatial confinement of photons both in the vertical and lateral directions, the carrier relaxation is accelerated to the bottom of the dispersion, which typically hinders the probing of the exciton–photon interaction by angle-resolved photoluminescence (PL) or reflectivity spectroscopy in such a geometry^34–36^. Fortunately, our mesa sizes are large enough compared to the wavelength of photons, such that the leaking electromagnetic field of light out of the mesa cavity is negligible and thus the exciton–photon coupling strength is almost the same as that of the planar microcavity^46^. The angle-resolved PL spectrum from a planar region (Fig. 1d) is measured as a reliable reference to characterize the exciton-photon interaction in the mesa cavity. Our results show that due to the strong coupling between excitons (*X*) and cavity (*C*) photons, the dispersion exhibits an anti-crossing behavior and is split into the lower polariton branch (LPB) and the upper polariton branch (UPB). By fitting the dispersion with a coupled oscillator model (S2 in Supporting Information), the exciton-photon detuning is extracted to be about $\delta ={E}_{C}-{E}_{X}=-75\,{\rm{meV}}$ and the Rabi splitting $\varOmega$ is ~37 meV, which satisfies the criterion for the strong coupling regime and confirms the formation of polaritons^18^. Meanwhile, the excitonic and photonic fractions (Fig. 1e) in the LPB can be obtained from the equations:

$$|X({k}_{//})\Big|^{2}=\frac{1}{2}\left[1+\frac{\delta ({k}_{//})}{\sqrt{{\varOmega }^{2}+\delta {({k}_{//})}^{2}}}\right],\,\Big|C({k}_{//})|^{2}=1-{|X({k}_{//})|}^{2}$$

where $X({k}_{//})$ and $C({k}_{//})$ are the Hopfield coefficients of exciton and photon, respectively. To simulate the confinement effect of polariton, the dispersion for a cylindrical mesa with a diameter of 4.5 μm is calculated by solving the coupled Schrödinger equation (S2 in Supporting Information) using the above experimental parameters. Assuming the Boltzmann distribution of polaritons^35,47^, the simulated dispersion can be obtained as shown in Fig. 1f. As a result of the lateral confinement provided by the mesa structure, it exhibits discrete energy levels, where the first three polariton states (*s*, *p*, and *d* state) can be clearly resolved.

**Fig. 2:**
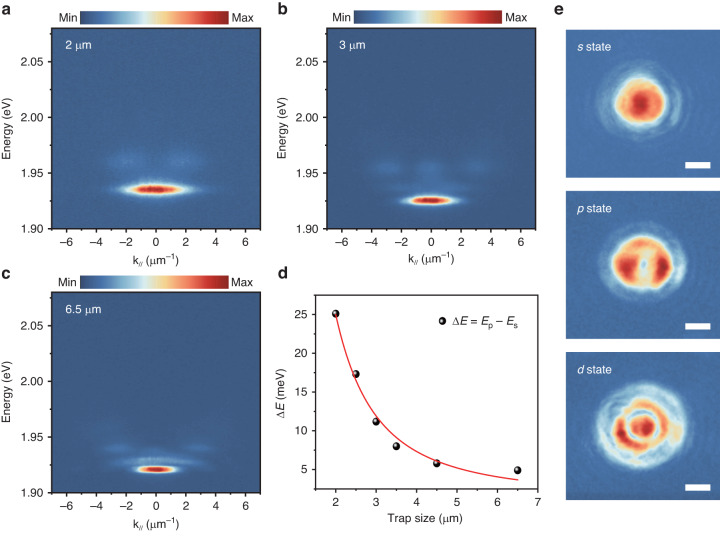
The dispersion and real-space distribution of trapped polariton. **a**–**c** Polariton dispersion map from 2 μm, 3 μm, and 6.5 μm mesa cavities. **d** The extracted energy spacing (black dot) between the *p* state $({E}_{p})$ and the *s* state $({E}_{s})$ versus the trap size. The red line represents the fitting results. **e** The real-space distributions of polariton emission at the energies of the *s*, *p*, an*d d* states in a 3 μm trap. The scale bar is 1 μm

To experimentally demonstrate the polaritons trapped by the mesa cavity, we directly mapped the dispersion and the spatial distribution of polaritons. Under a non-resonant excitation of continuous-wave (CW) laser at 532 nm, the angle-resolved PL spectra from the mesa cavity with diameters of 2 μm, 3 μm, and 6.5 μm are shown in Fig. 2a–c. All the maps show discrete energy levels, which is consistent with the calculated results in Fig. 1f. With the decrease of the trap size, the in-plane momentum gradually expands due to the Heisenberg uncertainty relation. At the same time, the energy spacing between the *p* state and *s* state increases. In fact, for the trapped particles in a cylindrical potential well, the energy levels ${E}_{n,k}$ can be described by: ${E}_{n,k}=\frac{{\hbar}^{2}}{2m}{(\frac{{q}_{n,k}}{R})}^{2}$, where *R* is the radius of the trap and ${q}_{n,k}$ represents the Bessel function with *n* and *k* being the quantum number^48^. Therefore, the energy difference between the *s* state and *p* state is expected to be inversely proportional to the squared trap radius. As shown in Fig. 2d, the experimental results are fitted with the inverse square function (red line), in excellent agreement with the theoretical expectation. In our 2 μm mesa cavity, the emission from *d*-state polaritons is too weak to be visible due to a large energy spacing relative to the ground state (greater than the thermal fluctuation of ~26 meV at room temperature). To visualize the real-space distribution of trapped polariton, the states corresponding to the energy bands of *s*, *p*, and *d* are extracted for imaging as shown in Fig. 2e. As expected, *s* and *p* states respectively display a cylindrically symmetric spot and a ring-like distribution, and the *d*-state has a combined character consisting of a spot and a ring-like profile. These observations agree with the photonic mode in a cylindrical potential well^48^, except for a slight deviation of intensity distribution from the ideal model, which may arise from the unintended strain or wrinkle in the sample^36^.

**Fig. 3:**
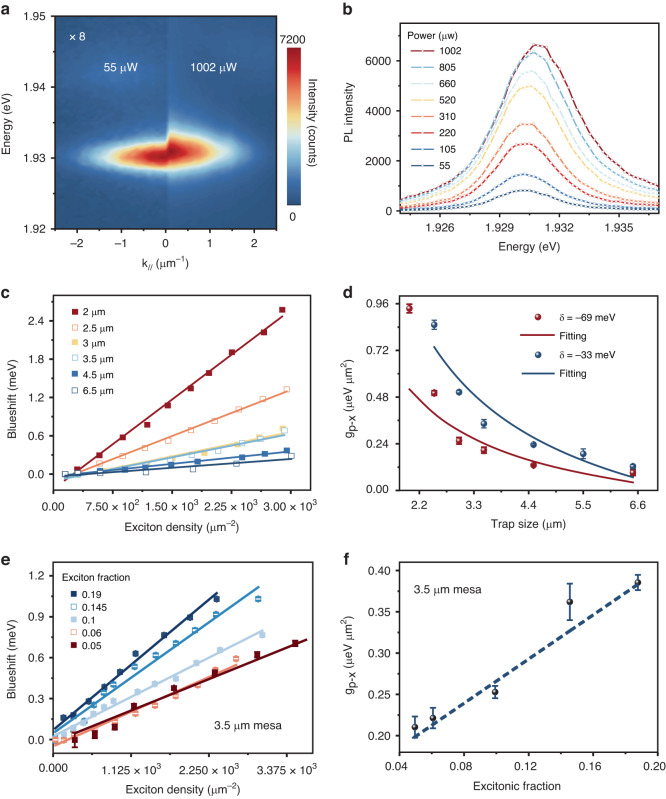
Manipulating the nonlinearity by trapping the exciton–polaritons. **a** Angle-resolved PL spectrum at low (left) and high (right) pump power for the 3 μm trap. The intensity in the left part is magnified by 8 for comparison. **b** Polariton spectra at ${k}_{//}=0$ for different excitation powers. **c** Blueshifts of *s*-state energies as a function of exciton density for varied trap sizes from 2 μm to 6.5 μm. **d** The estimated polariton–exciton interaction strength ${g}_{p-x}$ versus trap size at the detuning of $-33\,{\rm{meV}}$ (blue) and $-69\,{\rm{meV}}$ (red). **e** Blueshift*s* of *s* state as a function of exciton density with different exciton fractions. **f** Deduced interaction strength ${g}_{p-x}$ from (**e**) as a function of exciton fraction $({|X|}^{2})$. The dashed blue line is the linear fitting result

In order to investigate the polaritonic nonlinearity influenced by the spatial trapping, we measured the angle-resolved PL spectra when varying the incidence power of the CW laser at ambient conditions (see Materials and Methods). As shown in Fig. 3a, the *s*-state emission of the trapped polariton at 1002 μW has a noticeable blueshift compared with that at 55 μW in a 3 μm mesa cavity with a detuning of $-69\,{\rm{meV}}$. To quantitatively study the energy shift of the *s* state, the power-dependent spectra at ${k}_{//}=0$ were extracted and presented in Fig. 3b. Fitted by the Voigt function, the peak positions, intensities, and linewidths of all the spectra can be obtained (Fig. S2). It can be seen that increasing the pump power, the polariton peak shows a gradual blueshift.

We now investigate the possible attribution to the observed blueshift of the *s* polariton state. Under the CW non-resonant pumping in our experiment, the mean-field approximation predicts the energy shift^18^:

$$\varDelta E={g}_{p-p}{N}_{p}+{g}_{p-x}{N}_{x}$$

where ${g}_{p-p}({g}_{p-x})$ is the polariton–polariton (polariton-reservoir) interaction strength and ${N}_{p}({N}_{x})$ represents the polariton (exciton) density. In TMD microcavity, both effects from the exciton–exciton Coulomb interaction and the phase space filling contribute to polariton–polariton interaction strength^9^. The polariton-reservoir interaction constant ${g}_{p-x}$ is proportional to the exciton fraction in the polariton state.

To figure out the underlying mechanism of the observed nonlinear spectral response, we estimate the ratio of excitons to polaritons in our configuration with considering the Boltzmann distribution and the density of states of two species^49,50^:

$$\,\frac{{N}_{x}}{{N}_{p}}={e}^{-\varDelta E/{k}_{B}T}\frac{{D}_{ex}(E)}{{D}_{LP}(E)}={e}^{-\varDelta E/{k}_{B}T}\frac{{m}_{ex}}{{m}_{LP}}\approx {e}^{-\varDelta E/{k}_{B}T}\frac{{m}_{ex}}{{m}_{cav}/{|C|}^{2}}$$

where ${m}_{ex}$, ${m}_{cav}$, and ${m}_{LP}$ are the effective masses of bare excitons, cavity photons, and LPB, respectively. $\varDelta E$ is the energy splitting between the polariton ground state and the bare exciton state, which is approximately $-69\,{\rm{meV}}$. Typically, ${m}_{cav}$ is of the order of ${10}^{-4}{m}_{ex}$^18^, giving rise to a ratio of around 660 between the exciton and polariton populations at room temperature. Thus, under our nonresonant optical pumping condition, the polariton–exciton nonlinear interaction dominates in the blueshift, resulting in the energy shift being approximately linear to the exciton density, i.e., $\varDelta E={g}_{p-x}{N}_{x}$.

Next, to determine the polariton–exciton interaction strength, we first calculated the exciton density under different excitation power (S4 in Supporting Information). Then the polariton peaks were plotted as the orange solid squares in Fig. 3c, showing a linear increase with the exciton density. To further reveal the spectral response to the trapping size, the blueshifts of the peak position in different mesa sizes were also plotted for comparison. It clearly indicates that all peak positions of *s* states are linearly shifted with the exciton density, but the smaller the mesa cavity is, the more the peak shifts, implying the polariton–exciton interactions can be significantly enhanced by increasing the spatial confinement^16,28,51^. At the detuning of $\delta =-69\,{\rm{meV}}$, the polariton–exciton interaction strength, that is, the slope for the exciton-density-dependent energy shift, is extracted and plotted in Fig. 3d as the red dots. Theoretically, in the presence of micrometer lateral confinement, the cylindrical mesa cavities exclusively trap the polariton through the photonic part and hardly affect the exciton–exciton interaction due to its much shorter Bohr radius (~1 nm). Therefore, the wave function distribution of polariton varies with the trap sizes (Fig. S7a), which determines the exciton-polariton spatial overlap and thus the interaction coefficient ${g}_{p-x}$. To further quantify the ${g}_{p-x}$ as a function of trap sizes, the spatially integral function between exciton and polariton distribution is calculated (Fig. S7). It is worth mentioning that due to the short diffusion length of excitons (472 nm measured by the steady-state PL imaging technique in Fig. S3), their spatial distribution almost keeps unchanged for different trap dimensions and is directly denoted by the real-space profile of PL intensity. As for the polariton, its distribution can be characterized by the simulated *s*-state polariton mode in different mesas. The calculated result indicates that the ${g}_{p-x}$ is related to the trap diameter *d* by ${g}_{p-x}\propto {d}^{-0.73}$ (S5 in Supporting Information), which is consistent with the experimental data in Fig. 3d and shown as the fitting lines. To further reveal the controllable nonlinearity by confining polaritons, we resonantly excite the mesa cavities using a pulse laser and analyze the shift of polariton peaks, obtaining the trap-size-dependent polariton–polariton interaction strength (Fig. S5, 6). All the experimental results show that artificially creating potential landscapes is an encouraging strategy for enhancing polariton–exciton interaction strengths by about six times and polariton–polariton interaction strengths by one order of magnitude. We note that in 2 μm mesa, the measured ${g}_{p-x}$ deviates from the calculated result, which may be because the polariton–polariton interaction is inversely proportional to the square of the trap size^52,53^ and has an observable contribution to the spectral shift in such tight confinement. To further examine the feasibility of manipulating nonlinear interaction, we also performed the same analyses in the mesa cavities with a detuning of $-33\,{\rm{meV}}$ (blue dots in Fig. 3d), and ${g}_{p-x}$ also shows a dramatic increase with the decreasing trapping size, which obeys a similar rule to that at the $-69\,{\rm{meV}}$ detuning. Furthermore, since the polaritons have more excitonic fractions in the mesa cavity with $-33\,{\rm{meV}}$ detuning, ${g}_{p-x}$ exhibits larger values accordingly. To elaborate on the effect of exciton component on the polariton nonlinearity, we analyzed the power-dependent spectral shift (Fig. 3e) and ${g}_{p-x}$ as a function of exciton fractions (Fig. 3f) by fixing the trapping size at 3.5 μm. The interaction strength ${g}_{p-x}$ manifests a linear increase with the exciton fraction, further corroborating the hypothesis that the polariton–exciton nonlinear interaction dominates in the blueshift^54^. Thus, these results demonstrate that the spatial confinement by an artificial potential well can be employed to manipulate the nonlinearity of exciton polaritons at room temperature.

**Fig. 4:**
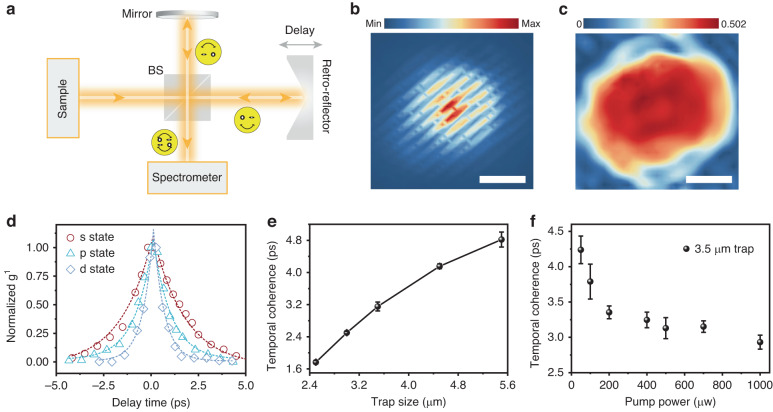
Controllable macroscopic coherence of trapped exciton–polaritons. **a** Schematic description of Michelson interferometer for the interference fringes measurement. BS for beam splitter. **b** Interference image of *s*-state polariton emission at zero time delay for the mesa of 3.5 μm. The scale bar is 1 μm. **c** The corresponding first-order correlation function ${g}^{1}(\tau =0)$ through the Fourier transform of (**b**). **d** Normalized ${g}^{1}$ varying with the time delay between the interference arms for *s*-, *p*-, an*d d-*state polaritons. The experimental data (hollow geometries) are fitted (dashed line) with exponential functions. **e** Coherence time as a function of trap size in the cavity. The error bars correspond to the exponential fitting uncertainty. **f** Power-dependent coherence time of polariton in a 3.5 μm trap

In addition to the nonlinearity, polaritons also possess the features of long-range propagation and macroscopic coherence from the view of the photonic side^9,34,36^. Phase coherence can be characterized by the first-order coherence function ${g}^{1}$, which can be degraded by energy fluctuation of the concerned polariton states^55^. In this section, we probe and analyze the phase coherence of the *s*-, *p*-, and *d*-state polaritons in the artificial mesa traps. As shown in Fig. 4a, a modified Michelson interferometer with a delay line allows us to investigate the coherence in the time domain (see Materials and Methods). A representative interference image from *s*-state polaritonic emission in a 3.5 μm trap is shown in Fig. 4b at zero temporal delay (see Fig. S8 for the *p*- and *d*-state interference), and a clear fringes contrast can be observed. Figure 4c is the corresponding coherence function ${g}^{1}(\tau =0)$ map through the Fourier transform of the interferograms in Fig. 4b. The maximum ${g}^{1}(\tau =0)$ reaches a value of 0.502 for the in-situ interference of the *s*-state polaritons. To investigate the coherence time, we systematically record the ${g}^{1}$ distributions versus the time delay for three different polariton modes, and the normalized results are plotted in Fig. 4d. It can be found that the ${g}^{1}$ decay more quickly for the higher-energy state, which may be attributed to the reduced photon/exciton ratio in the polaritons, leading to the less intrinsic coherence and faster interaction-induced dephasing. Different from the Gaussian decay in the previous report^35^, the time-dependent ${g}^{1}(\tau )$ here was fitted by exponential functions considering the slower coherence decay than the polariton dissipation (S8 in Supporting Information). As a result, the coherence time (1/*e* of the exponential decay) of 3.15 ± 0.13 ps for *s*-state polaritons was extracted. For *p*- and *d*-state polaritons, the coherence time is shortened to 1.92 ± 0.08 ps and 1.15 ± 0.04 ps, respectively.

It is worth emphasizing that compared with the free polaritons, spatial trapping leads to the suppression of inhomogeneous broadening and spectral linewidth narrowing and thus enhances the macroscopic coherence for the trapped polaritons^34,36^. In contrast, the increased polariton-reservoir interaction for the tighter confinement, as demonstrated above, would accelerate the decoherence rate of polaritons and shorten the coherence time. Under CW excitation at 532 nm, we extract the coherence time of *s*-state polariton versus the trap size of mesa cavities and find a dramatic reduction of coherence time when decreasing the trap size as shown in Fig. 4e. Correspondingly, a broadened linewidth of *s*-state polaritons was observed in the smaller trap under same excitation condition (Fig. S13), which is also evidence for the increased incoherent scattering rate driven by the polariton–exciton interaction^49^. To further examine the universality, we measured another two samples with different detuning and observed a similar trend (Fig. S9). Therefore, the interaction-induced dephasing plays a dominant role in the coherence time of trapped polaritons. It can be anticipated that when the interaction-induced dephasing is relieved and the suppression of inhomogeneous broadening by the trap still works in a larger trap, a maximum coherence of trapped polaritons can be found. However, the energy difference between *s* and *p* states gets smaller in larger traps (Fig. 2d), hindering further exploration in experiement. According to the trend of the increased temporal coherent with the trap sizes and the fact of the ultrafast decoherence time (542 fs) for free polariton (Fig. S14), there must be an optimal confinement for the longest coherence and needs more theoretical analyses beyond the scope of our study.

Based on the above results, managing the existing excitons in the reservoir becomes an effective strategy to manipulate the coherence, and also provides a robust criterion for distinguishing the emission from the strong or weak interaction regime^56^. Hence, we measure the phase coherence of *s*-state polaritons at variable pump powers in a trap (3.5 μm). As shown in Fig. 4f, the coherence time has a rapid reduction below the power of 200 μW and slowly decreases with the pump power due to the increased polariton–exciton collision (Fig. S2). This behavior is different from pure photonic emission whose coherence shows an opposite evolution with the pump power. So these results imply that our system is in a strong-interaction regime and particle-pairs interaction contributes to the polariton dephasing (another sample shown in Fig. S10). Furthermore, the power-dependent intensity and linewidth don’t show a clear threshold-like inflection point (Fig. S2), so the increase of temporal coherence for the trapped polariton is attributed to the suppression of spectral linewidth rather than the polariton lasing^16,34^. All the above analyses together suggest that our mesa cavities are a versatile platform for engineering the polariton coherence properties.

## Discussion

In conclusion, we have presented a robust strategy for manipulating the nonlinear interaction and the macroscopic coherence of polaritons in monolayer WS_2_ through the artificial potential landscapes at room temperature. The angle-resolved PL spectra and real-space image of polaritons unambiguously demonstrate the trapping effect provided by the mesa cavities, whose sizes determine the nonlinear polariton–exciton interaction strength and thus the coherence time of polaritons. Shrinking down the mesa from 6.5 μm to 2 μm, the polariton–exciton interaction constant is enhanced by about 6 times. Meanwhile, the coherence time of polaritons is dramatically extended due to spectra narrowing^34^ and tuned in several-picosecond ranges through defining the spatial trapping. Therefore, these results prove a convenient method based on the programmed micro-nano fabrication to achieve controllable nonlinearity and coherence of polaritons in TMD at room temperatures, which lay the foundation for simulating the polariton Hamiltonian with further complex potential landscapes, such as Su-Schrieffer-Heeger model^32,33^, Kagome lattices^42,57^, and other topological structures^31,58^. We believe that this work represents an important milestone and would stimulate more developments in realistic polaritonic applications based on the TMD microcavities, such as polariton modulators, polariton quantum sources, and quantum information processing^19,37,59^.

## Materials and methods

### Optical measurement

The PL spectra were excited by a CW laser (532 nm) focused by a $100\times$ objective (Nikon, N.A. = 0.9). The angle-resolved reflection and PL measurements were performed by projecting the back focal plane of the objective onto a spectrometer (iHR 550) equipped with a charge-coupled device. To resolve the peak energy shift in the nonlinear measurement, a grating with high-resolution (600 lines/mm) was employed. The time-dependent interference images were obtained using a modified Michelson interferometer with a retro-reflector on a uniaxial delay stage. The images from two interferent arms were center-inversion and spatially overlapped on the charge-coupled device. A set of tunable long- and short-pass filters (Semrock) were used to extract the interested energy from the trapped polariton state.

### Sample fabrication

The monolayer WS_2_ was mechanically exfoliated from bulk crystal (HQ Graphene) and then transferred onto the bottom DBR (Reflectance >99.99% at *λ* = 620 nm) consisting of 16 pairs of SiO_2_/TiO_2_ layers. A few layers of hBN were used to protect the WS_2_ from damage in the subsequent fabrication process. Then 108-nm-thick PMMA was spin-coated both as the cavity spacer and exposure layer, which was patterned into a series of cylindrical mesas with electron-beam lithography. The diameters of the mesa range from 2 μm to 6.5 μm and a distance of more than 2.5 μm between neighboring structures was adopted to avoid the cross-interaction. Finally, the top DBR with 8.5 pairs of TiO_2_/SiO_2_ layers was deposited by the electron-beam evaporator to construct the cavity.

### Supplementary information

supporting materials for publication
