# Supplementary material for: Manipulating nonlinear exciton polaritons in an atomically-thin semiconductor with artificial potential landscapes
Source: Light Sci Appl. 2023 Sep 8;12:220. doi: 10.1038/s41377-023-01268-2 (PMC10485014; doi:10.1038/s41377-023-01268-2)
Supplement: Supplementary file 1 — supporting materials for publication [file 41377_2023_1268_MOESM1_ESM.pdf]

Supplementary Information for

Manipulating nonlinear exciton polaritons in an atomically-thin semiconductor with artificial potential landscapes

Yuan Luo[1,†], Quanbing Guo[2,†], Xinyi Deng[3], Sanjib Ghosh[4], Qing Zhang[3], Hongxing Xu[2,5,*], and Qihua Xiong[1,4,6,7,*]

[1]State Key Laboratory of Low-Dimensional Quantum Physics, Department of Physics, Tsinghua University, Beijing 100084, China
[2]Wuhan Institute of Quantum Technology, Wuhan 430206, China
[3]School of Materials Science and Engineering, Peking University, Beijing 100871, China
[4]Beijing Academy of Quantum Information Sciences, Beijing 100193, China
[5]School of Physics and Technology, Center for Nanoscience and Nanotechnology, and Key Laboratory of Artificial Micro- and Nano-structures of Ministry of Education, Wuhan University, Wuhan 430072, China
[6]Frontier Science Center for Quantum Information, Beijing 100084, China
[7]Collaborative Innovation Center of Quantum Matter, Beijing, China

[†]These authors contributed to this work equally.

To whom the correspondence should be addressed. Email: qihua_xiong@tsinghua.edu.cn, and hxxu@whu.edu.cn

S1 Characterization of the empty mesa and planar cavities

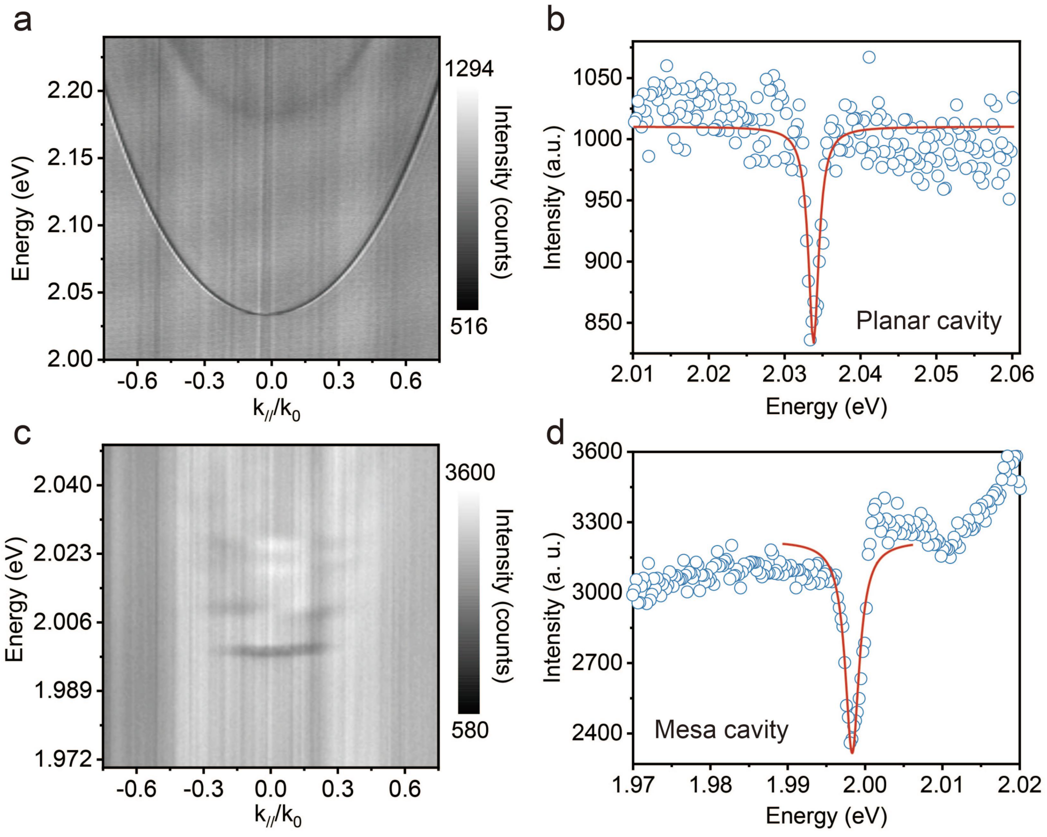

Fig. S1 (a) Angle-resolved reflection map of the bare cavity mode measured on the planar microcavity, showing a blueshift compared with that in hBN/WS_2 area. (b) Extracted spectrum at $k_{//}=0$ to calculate the Q factor. Through Lorentzian fitting, the resonance peak is at 2.03381 eV with an FWHM of ~1.67 meV. The quality factor is Q = 1218 ± 167. (c) Dispersion map of bare mesa cavity. The discrete photonic mode can be clearly resolved in the LPB. (d) Spectrum at $k_{//}=0$ in the bare mesa cavity with Q = 896 ± 100.

S2 Coupled oscillator model and simulation of trapped polariton state in a potential

Exciton polariton can be formed when the interaction between semiconductor excitons and cavity photons is faster than their individual decay rates. For a monolayer WS_2 in a planar cavity, the two hybridized polariton branches, *i.e.*, the

LPB and UPB can be described by coupled oscillator model[1]:

$$E_{LP/UP}\left(k_{//}\right)=\frac{1}{2}\left[E_X\left(k_{//}\right)+E_C\left(k_{//}\right)\mp\sqrt{\Omega^2+\delta^2\left(k_{//}\right)}\right]$$

Where $k_{//}$ represents the in-plane wavevector, $E_X\left(E_C\right)$ is the energy of the exciton resonance (cavity mode), δ is the detuning defined as $\delta=E_C-E_X$, and Ω is the Rabi splitting. The cavity mode can be expressed: $E_C\left(k_{//}\right)=E_C\left(0\right)+\hbar^2k_{//}^2/2m_C$, where m_C is the effective mass of the bare cavity, which is typically on the order of 10^{-4} m_{exc} [1]. With the known Hopfield coefficients $|C|$ and $|X|$ in the polariton branch, the effective mass and dispersion of LPB and UPB polaritons can be expressed as:

$$\begin{aligned} m_{LP}\left(k_{//}\right) &\simeq m_{cav}/|C|^2 \\ m_{UP}\left(k_{//}\right) &\simeq m_{cav}/|X|^2 \\ E_{LP/UP}\left(k_{//}\right) &= E_{LP/UP}\left(0\right)+\hbar^2k_{//}^2/2m_{LP/UP} \end{aligned}$$

To simulate the discrete polariton state in pillar microcavity shown in Fig. 1f, we calculate the dispersion relation via solving Schrodinger's equation[2] for polaritons in cylindrical mesa structures:

$$i\hbar\frac{\partial}{\partial t}\begin{pmatrix}\Psi_X\\ \Psi_C\end{pmatrix}=\begin{pmatrix}E_X & \Omega/2\\ \Omega/2 & E_C\left(0\right)+\dfrac{\hbar^2k_{//}^2}{2m_C}+V\left(\mathbf{r}\right)\end{pmatrix}\begin{pmatrix}\Psi_X\\ \Psi_C\end{pmatrix}$$

where the trapping of polaritons is achieved by confining their photonic part Ψ_C rather than their excitonic part Ψ_X. The confinement potential $V\left(\mathbf{r}\right)$ has the same profile as the cylindrical mesa structure with a finite depth of 183 meV (determined by atomic force microscope image shown in Fig. 1c). The time-independent eigenstate and eigenvalue can be obtained by separating the time-dependent evolution terms. And then we use Finite Difference Method (FDM) to calculate the matrix in real space assuming the Boltzmann distribution of polaritons[3].

S3 Intensity and linewidth to polariton spectrum at $k_{//} = 0$ for different pump powers

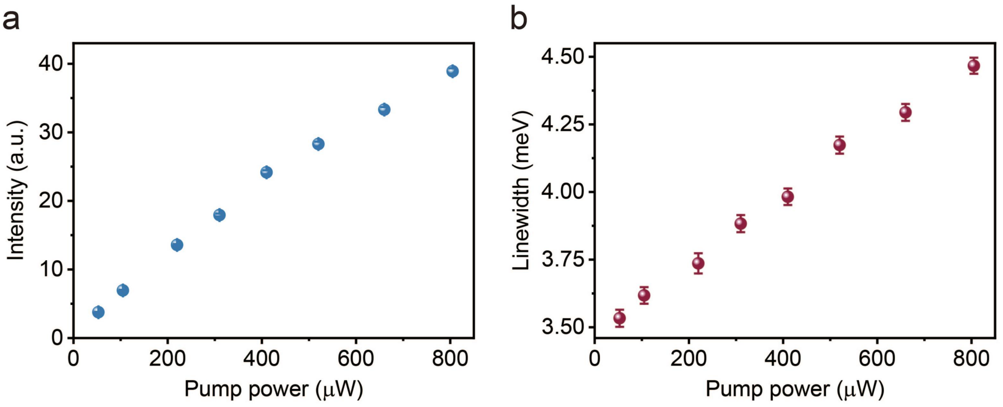

Fig. S2 (a) PL intensity and (b) linewidth of polaritons at $k_{//} = 0$ as a function of pump power for the mesa size of 3 μm. They both linearly increase with pump powers, indicating that the excitation is far below saturation to avoid bleaching of exciton-photon interaction[4].

S4 Estimation of exciton density and measuring the polariton nonlinearity by resonant reflectivity spectra

A. Obtaining the diffusion length and exciton density in the mesa cavity

To experimentally determine the exciton diffusion length in our system, we measure the imaging of the excitation spot (Fig. S3a) and the steady-state photoluminance (Fig. S3b) for the heterostructure hBN/WS_2 on the bottom DBR, which can be considered as the convolution between the laser's gaussian profile and the diffusion-length-dependent Bessel function K_0, that is,

$$n(r) \propto \int_{-\infty}^{\infty} K_0\left(r' / L_X\right) e^{-2(r-r')^2/w^2} dr'$$

where n(r) is exciton distribution, Lx is the exciton diffusion length, and w is related to the excitation profile (w = 486 nm). As shown in Fig. S3c, the PL linewidth is broader than that of the laser due to the exciton diffusion, and the diffusion length is fitted to be 0.472 μm, coincident with the values reported in previous literature[5,6]. Those results indicate that the excitons almost localize in the mesa region, and the

spatial distribution or effective density of excitons keeps unchanged for different trap dimensions. So the exciton distribution area is all the same in different mesa cavities.

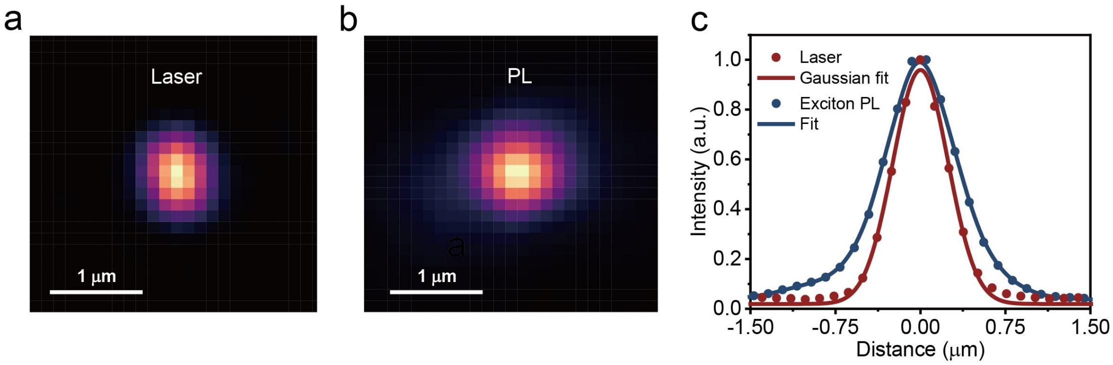

Fig. S3 (a) The real-space image of the excitation spot. (b) The PL image for the heterostructure hBN/WS_2 on the bottom DBR substrate. (c) Excitation and PL intensity profiles (dots) extracted from (a) and (b), and their corresponding fitting curves (lines).

For calculating the exciton-polaritons interaction strength in the trapping mesa cavities, we need to estimate the exciton density under the excitation of a 532 nm CW laser. The effective 2D exciton density n_x^{eff} is represented by[7]:

$$n_x^{eff} = \frac{P_{pump}\alpha\left(E_{pump}\right)}{S}\frac{\tau_X}{E_X}$$

where P_{pump} is the time-averaged pump power in the microcavity after considering the transmission coefficient of the cavity. S is the exciton distribution area and α represents the absorption of WS_2 at 532 nm ($\alpha\left(E_{pump}\right)\approx 3.3\%$)[8]. To determine the exciton lifetime in our system, we performed the time-resolved photoluminescence measurement for the heterostructure hBN/WS_2 on the bottom DBR with PMMA coated. With a 532 nm laser (pulse width of 6 ps with a repetition rate of 5 MHz), the exciton density is about 1.25×10^3 μm^{-2} and the exciton lifetime is deduced to be 233 ps (Fig. S4). To calculate the exciton distribution area, we used the width corresponding to the e^{-2} of the PL image profile as the exciton diameter. Then the S is

calculated to be 1.41 μm^2 after considering the exciton diffusion. Our calculation method results in effective 2D exciton densities from 10^2 to 10^3 μm^{-2}, far below the Mott density of the monolayer TMD[9].

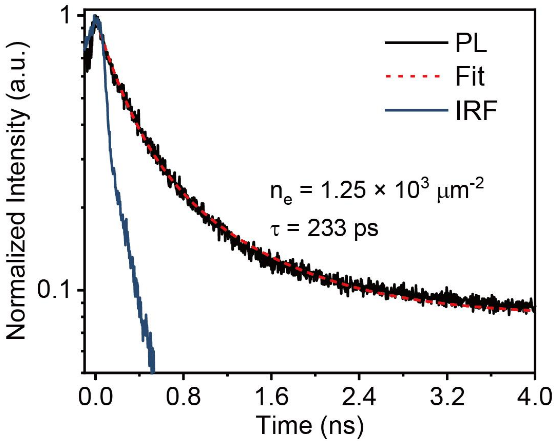

Fig. S4 Time-resolved photoluminescence (dark curve) fitted with an exponential decay model.

B. The dependence of nonlinear interaction constant of polariton on trapping size by resonant reflectivity measurement

In this section, we measured the nonlinear response of the artificial mesa cavities by resonant excitation to further reveal the controllable nonlinearity by confining polarions. To improve the depth of the reflection dips for the resonant measurement, we redesigned the mesa cavity with reduced pairs of SiO_2/TiO_2 for both the top and bottom DBR and utilized a femtosecond laser with a duration of about 145 fs and a repetition rate of 5 kHz to resonantly pump the system[10].

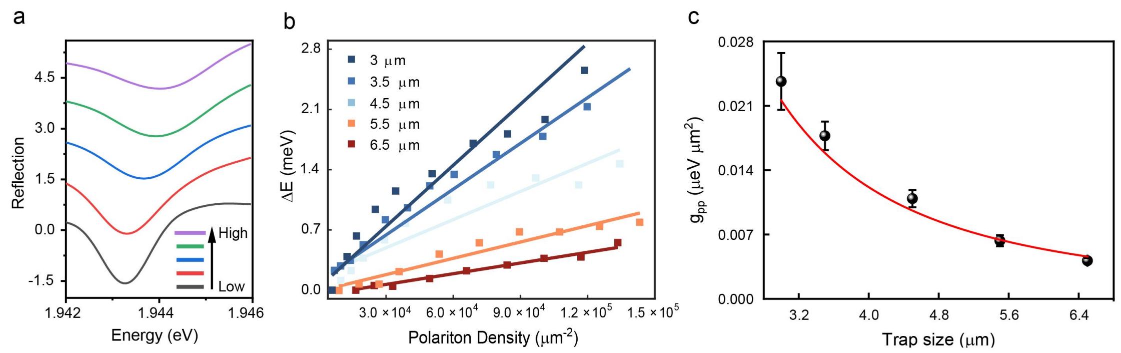

Fig. S5 (a) Polariton spectra at $k_{//}=0$ for different excitation powers in 5.5 μm mesa cavity, showing the blueshift from low pump fluence to high pump fluence. (b) Blueshift of s-state polariton as a function of polariton density for varied trap sizes from 3 μm to 6.5 μm. (c) The estimated polariton-polariton interaction strength g_{pp} versus trap size.

Fig. S5a shows the blueshift from low pump fluence to high pump fluence where we resonantly excite the 5.5 μm pillar microcavity. To quantitatively analyse the polariton-polariton interaction, the polariton density has been calculated by the following equation[10,11]:

$$n=\frac{P\int_{k_{//}=0}G\left(E\right)Absor_{LP}(E)dE}{A_{beam}fE_{LP}\int G\left(E\right)dE}$$

where $G(E)=\frac{A_{laser}}{\gamma_{laser}\sqrt{\pi/2}}e^{-\frac{2(E-E_{laser})^2}{\gamma_{laser}^2}}$ represents the laser profile with a Gaussian shape, and $Absor_{LP}\left(E\right)$ is the absorption of the s-state polariton by fitting the reflection spectra. The P and f is the average power and repetition rate of the laser, respectively. $A_{beam}=0.65\ \mu\text{m}^2$ is the laser beam area. By fitting the reflection dips of the polariton with the Lorentzian function, we obtain the polariton energies plotted as the orange solid squares in Fig. S5b, showing a linear increase with the polariton density. To further reveal the nonlinear response to the trapping size, the blueshifts of the peak position in different mesa sizes with similar detuning were also plotted for

comparison, indicating that the smaller the mesa cavity is, the more the peak shifts. The polariton-polariton interaction, that is, the slope of the polariton-density-dependent energy shift, is extracted and plotted in Fig. S5c and fitted by an inversely quadratic function (red line), which is in agreement with the theoretical expectation[12]. Additionally, compared with a planar microcavity with similar detuning (Fig. S6), the value of polariton-polariton interaction of the mesa cavity shows an increase of nearly an order of magnitude. These results undoubtedly demonstate that confining polaritons can enhance the polariton-polariton interaction strength[13].

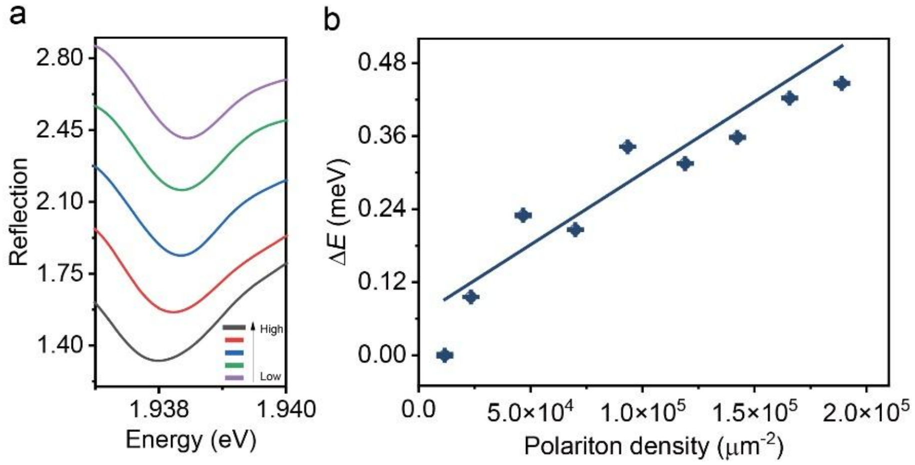

Fig. S6 Measurement of the polariton-polariton interaction in the planar cavity. (a) Reflection spectra of s-state polariton at $k_{//} = 0$ for different pumping densities. (b) The spectra dip energies extracted from (a) as a function of polariton density. The linear fitting result indicates the polariton-polariton interaction of $0.0023\ \mu\mathrm{eV}\cdot\mu\mathrm{m}^2$, in agreement with previous measurements[7]

S5 The theory about manipulating nonlinear interaction strength

In a mesa cavity, the polariton-exciton interaction Hamiltonian is given by:

$$H_I = g_{x-x} |X|^2 \int \Phi^+(\mathbf{r})\,\Phi\,(\mathbf{r})\Psi^+(\mathbf{r})\Psi\,(\mathbf{r})d\mathbf{r}$$

Where $\Phi(\mathbf{r})$ is the field operator of the cavity photons and $\Psi(\mathbf{r})$ is the field operator of the excitons. In the quantum description, we can express the Hamiltonian

as

$$H_I = g_{x-x} |X|^2 \sum_{nm} a_n^+ a_n c_m^+ c_m \int |\psi_n(r)|^2 |\varphi_m(r)|^2 d\mathbf{r}$$

Where $\Phi(\mathbf{r}) = \sum_n a_n \psi_n(\mathbf{r})$, and $\Psi(\mathbf{r}) = \sum_m c_m \varphi_m(\mathbf{r})$. The operators a_n and c_m are the annihilation operators of the cavity photons and excitons respectively. $\psi_n(\mathbf{r})$ and $\varphi_m(\mathbf{r})$ are the single-particle wave functions of the cavity photon and exciton, respectively. Under a mean-field approximation, we replace the exciton number operator $c_m^+ c_m$ with its expectation value $\langle c_m^+ c_m \rangle$. Furthermore, the exciton population follows a distribution function $P_x(\mathbf{r})$, such that we can write:

$$\sum_m |\varphi_m(\mathbf{r})|^2 \langle c_m^+ c_m \rangle \approx N_{tot} P_x(\mathbf{r})$$

where N_{tot} is the total number of excitons which relates to the exciton density as $N_x = N_{tot} / S$, and S is the exciton distribution area. Then

$$H_I = \left[N_x g_{x-x} |X|^2 S \int |\psi_n(\mathbf{r})|^2 P_x(\mathbf{r}) d\mathbf{r} \right] \sum_n a_n^+ a_n = \sum_n \Delta E_n a_n^+ a_n$$

We can now identify that the blueshift of a quantized mode (n-th mode) is given by

$$\Delta E = N_x g_{x-x} |X|^2 S \int |\psi_n(\mathbf{r})|^2 P_x(\mathbf{r}) d\mathbf{r}$$

When polariton-reservoir interaction dominates the spectral shift under nonresonant excitation ($\Delta E = g_{p-x} N_x$), we find that the effective polariton-exciton interaction strength for s-state polariton is given by:

$$g_{p-x} = g_{x-x} |X|^2 S \int |\psi_s(\mathbf{r})|^2 P_x(\mathbf{r}) d\mathbf{r}$$

Note that the exciton-exciton interaction strength g_{x-x} is unaffected by the trap size, since the trap size in our study is several micrometers, much larger than the excitonic Bohr radius but comparable with the polaritonic spatial extension. So, in the presence of confinement, although the exciton density is independent of the trap sizes, the spatial overlap between the exciton and polariton is changed in different-size traps, which is mainly due to the photonic component of polaritons confined by the mesas

and thus modifies the polariton-exciton interaction. In the calculation, the $\left|\psi_s(\mathbf{r})\right|^2$ and $P_x(\mathbf{r})$ can be represented by the spatial distributions of the simulated photonic mode and photoluminance, as shown in Fig. S7a. As a result, the g_{p-x} is dependent on the trap diameter d with a relationship of $g_{p-x} \propto d^{-0.73}$ (Fig. S7b), then we can achieve the manipulation of polariton- reservoir interaction through tuning the trap sizes. It is important to note that the polariton-reservoir interaction in essence is from the exciton-exciton interaction, among which one exciton strongly couples with cavity photon and the other exciton is from the reservoir[4,14-16].

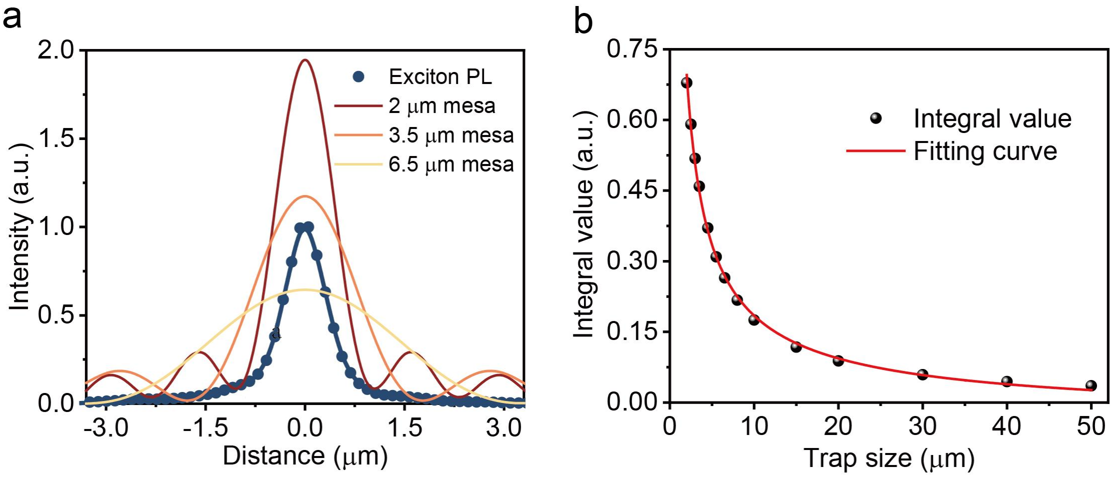

Fig. S7 (a) The spatial distribution of the experimental photoluminance and the simulated s-state polariton in different traps. (b) The integral value of spatial overlap as a function of cylindrical radius. The black dot is the calculated result and the red curve is the fitting result with a function of $d^{-0.73}$.

S6 The phase coherence of *p*- and *d*-state polaritons

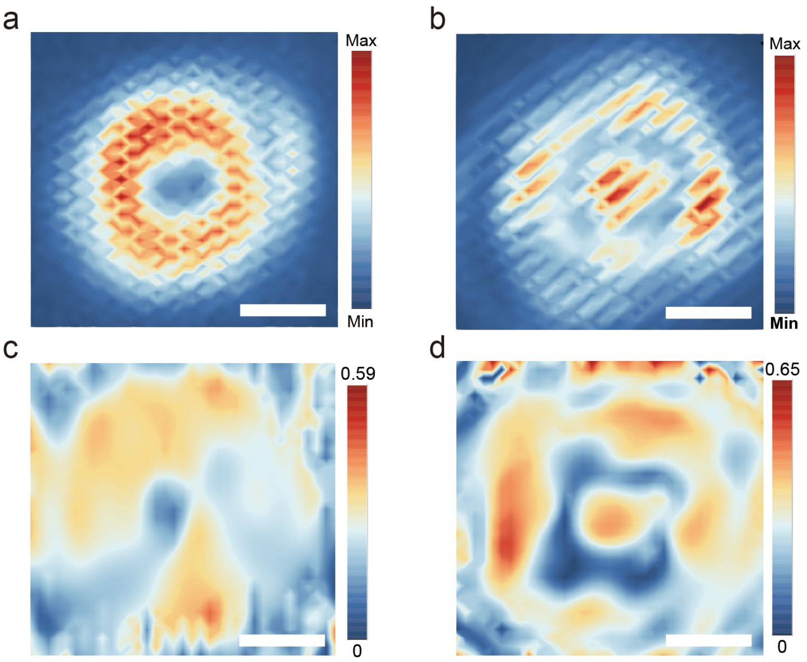

Fig. S8 (a)-(b) The inference image of *p*-state and *d*-state polariton at zero time delay for the mesa of 3.5 μm. The scale bar is 1 μm. (c)-(d) The corresponding first-order correlation function $g^1(\tau = 0)$ through the Fourier transform of (a)-(b), respectively.

S7 The phase coherence related to trap size and pump power

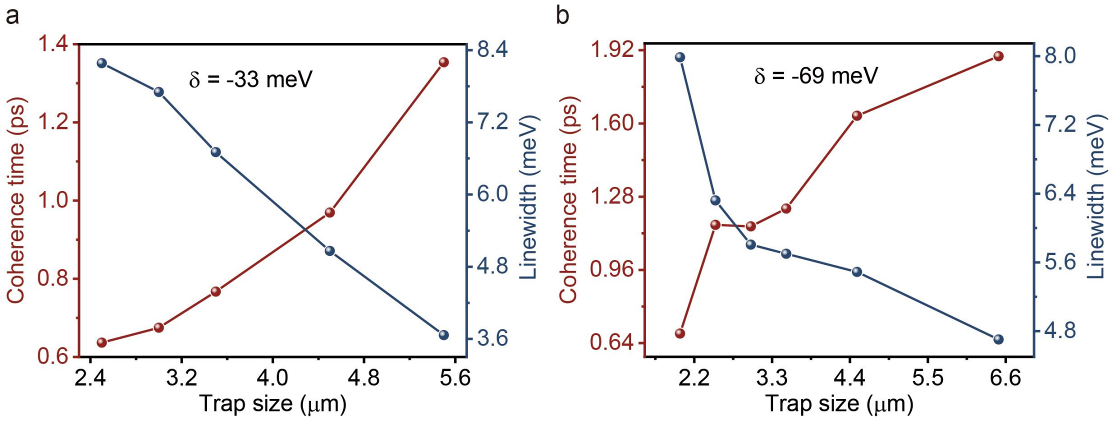

Fig. S9 The coherence time of *s*-state polaritons as a function of trap size in other mesa cavities at the detuning of (a) -33 meV and (b) -69 meV. Increasing the trap size, the polariton linewidth is narrowed and the coherence time becomes longer.

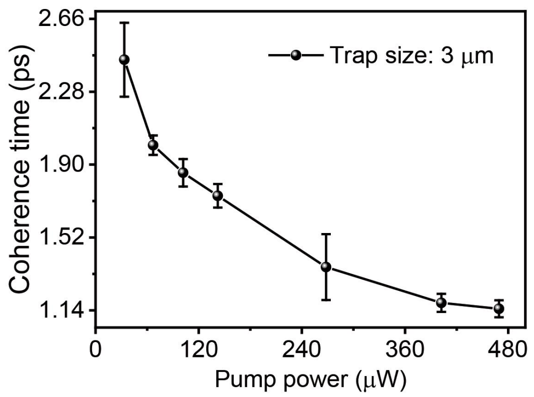

Fig. S10 Power-dependent coherence time of *s*-state polaritons in a 3 μm trap in another mesa cavity (not the studied one in Figure 4) at the detuning of -69 meV.

S8 Spectral narrowing and enhanced coherence of polaritons

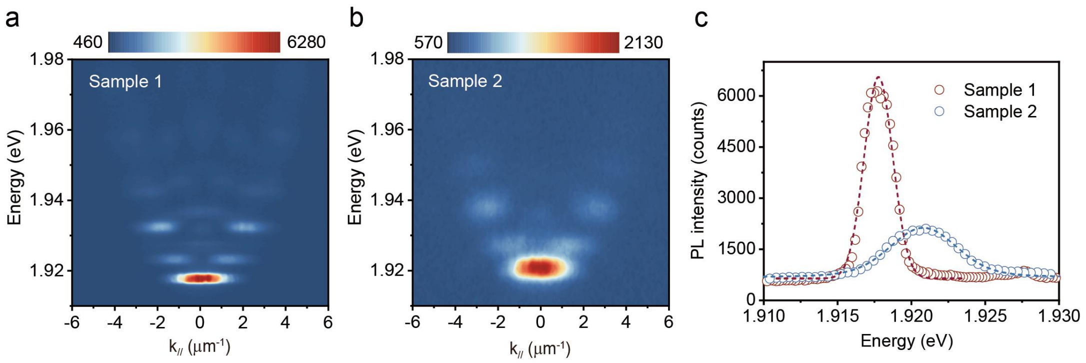

Fig. S11 The comparison of polariton dispersion with (a) narrow (sample 1, the studied one in the main text) and (b) broad (sample 2) linewidth in a 4.5 μm mesa cavity. (c) PL spectra from sample 1 (red dots) and sample 2 (blue dots) at $k_{//} = 0$. The experimental data are fitted with the Voigt function and the linewidth of 2.29 meV and 5.43 meV are obtained for sample 1 and sample 2, respectively.

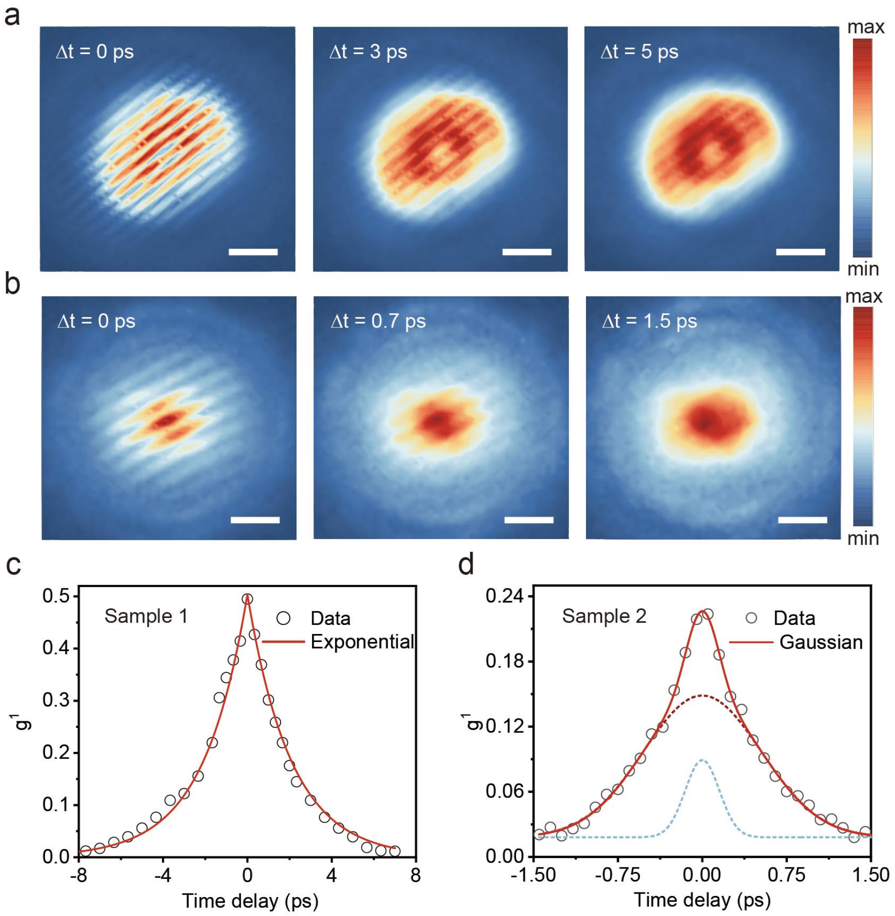

Fig. S12 The comparison of the macroscopic coherence in two different samples. (a) The polariton interference image from sample 1 at time delays $\Delta t = 0$ ps (left column), $\Delta t = 3$ ps (central column) and $\Delta t = 5$ ps (right column). (b) The polariton interference image from sample 2 at time delays $\Delta t = 0$ ps (left column), $\Delta t = 0.7$ ps (central column), and $\Delta t = 1.5$ ps (right column). (c) The first-order correlation function g^1 of the s-state polaritons in sample 1 as a function of time delay. The experimental results are represented by black circles, fitted with an exponential decay function. (d) The delayed first-order correlation function of the s-state polaritons from sample 2, which is fitted with two Gaussian functions. The red dashed line corresponds to the signal and the blue dashed line is the background from high-momentum polaritons[8].

To compare the macroscopic coherence of polaritons with different linewidth, we use the model about atom lasers with interactions proposed in Ref.[17] Through this model, the first-order correlation function can be expressed as:

$$
\begin{aligned}
|g^1(\tau)| &\approx g^1(0)\exp(-2u^2 n_c \tau^2)\exp(-\gamma\tau/2(n_c-n_s)) \qquad & (n_c-n_s)\gamma\tau/n_c \le 1 \\
&\approx g^1(0)\exp(-4u^2 n_c^2 \tau/((n_c-n_s)\gamma))\exp(-\gamma\tau/2(n_c-n_s)) \qquad & (n_c-n_s)\gamma\tau/n_c \gg 1
\end{aligned}
$$

where u represents polariton interaction and γ is the decay rate of ground-state polariton. n_c is related to the pump strength and n_s is the gain saturation number. For both cases, the last term represents the Schawlow-Towns formula which can describe the coherent characterization of the photon laser[18]. The middle term reveals the particle-pairs interaction leading to the dephasing of the polariton state. So when the coherence time is larger than the polariton lifetime, the first-order correlation function approaches an exponential form (sample 1). Otherwise, the first-order correlation function evolves to Gaussian form (sample 2).

S9 Linewidth of polariton spectrum at $k_{//}=0$ for different trap size

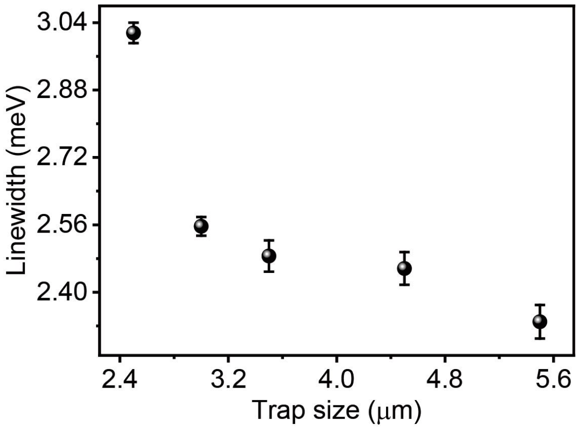

Fig. S13 Linewidth of *s*-state polaritons at $k_{//}=0$ as a function of trap size for the mesa cavities, showing an increase with decreasing the trap size.

S10 Macroscopic coherence of free exciton-polaritons

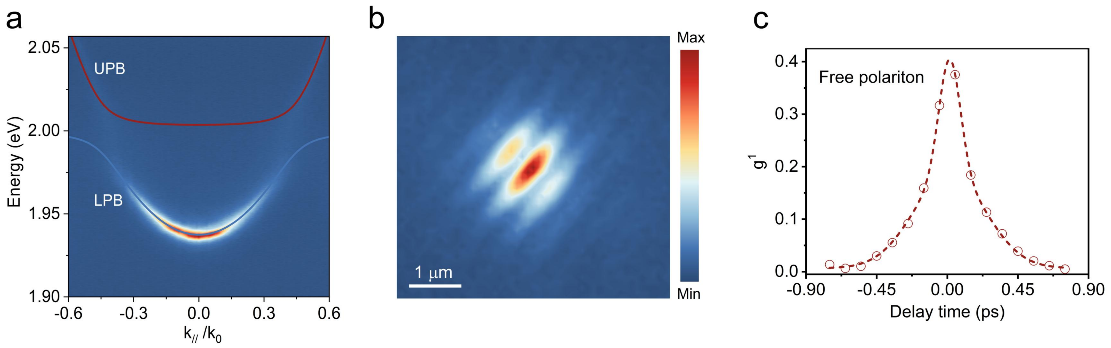

Fig. S14 (a) PL dispersion collected from a planar microcavity with a detuning of -62 meV. The red (blue) line represents the upper (lower) polariton branch. (b) Interference image of free polariton at zero time delay. (c) The corresponding first-order correlation function g^1 varying with the time delay. The experimental data (red circles) are fitted (dashed line) with two Gaussian functions, showing a much shorter temporal coherence (542 fs) than that in the trapped case.

Reference

1 Deng, H., Haug, H. & Yamamoto, Y. Exciton-polariton Bose-Einstein condensation. *Reviews of Modern Physics* **82**, 1489-1537 (2010).

2 Schneider, C. *et al.* Exciton-polariton trapping and potential landscape engineering. *Reports on Progress in Physics* **80**, 016503 (2017).

3 Shan, H. Y. *et al.* Spatial coherence of room-temperature monolayer WSe_2 exciton-polaritons in a trap. *Nature Communications* **12**, 6406 (2021).

4 Daskalakis, K. S. *et al.* Nonlinear interactions in an organic polariton condensate. *Nature Materials* **13**, 271-278 (2014).

5 Cadiz, F. *et al.* Exciton diffusion in WSe_2 monolayers embedded in a van der Waals heterostructure. *Applied Physics Letters* **112** (2018).

6 Goodman, A. J. *et al.* Substrate-Dependent Exciton Diffusion and Annihilation in Chemically Treated MoS_2 and WS_2. *Journal of Physical Chemistry C* **124**, 12175-12184 (2020).

7 Zhao, J. X. *et al.* Nonlinear polariton parametric emission in an atomically thin semiconductor based microcavity. *Nature Nanotechnology* **17**, 396-402 (2022).

8 Zhao, J. X. *et al.* Ultralow Threshold Polariton Condensate in a Monolayer Semiconductor Microcavity at Room Temperature. *Nano Letters* **21**, 3331-3339 (2021).

9 Anton-Solanas, C. *et al.* Bosonic condensation of exciton–polaritons in an atomically thin crystal. *Nature Materials* **20**, 1233-1239 (2021).
10 Zhang, L. *et al.* Van der Waals heterostructure polaritons with moire-induced nonlinearity. *Nature* **591**, 61-65 (2021).
11 Datta, B. *et al.* Highly nonlinear dipolar exciton-polaritons in bilayer MoS_2. *Nature Communications* **13**, 6341 (2022).
12 Verger, A., Ciuti, C. & Carusotto, I. Polariton quantum blockade in a photonic dot. *Physical Review B* **73**, 193306 (2006).
13 Delteil, A. *et al.* Towards polariton blockade of confined exciton–polaritons. *Nature Materials* **18**, 219-222 (2019).
14 Su, R. *et al.* Room-Temperature Polariton Lasing in All-Inorganic Perovskite Nanoplatelets. *Nano Letters* **17**, 3982-3988 (2017).
15 Bao, W. *et al.* Observation of Rydberg exciton polaritons and their condensate in a perovskite cavity. *Proceedings of the National Academy of Sciences of the United States of America* **116**, 20274-20279 (2019).
16 Wouters, M. & Carusotto, I. Excitations in a nonequilibrium Bose-Einstein condensate of exciton polaritons. *Physical Review Letters* **99**, 140402 (2007).
17 Love, A. P. D. *et al.* Intrinsic decoherence mechanisms in the microcavity polariton condensate. *Physical Review Letters* **101**, 067404 (2008).
18 Kim, S. *et al.* Coherent Polariton Laser. *Physical Review X* **6**, 011026 (2016).
